# Sustainable Food Systems and the Mediterranean Diet

**DOI:** 10.3390/nu11092229

**Published:** 2019-09-16

**Authors:** Elliot M Berry

**Affiliations:** Braun School of Public Health, Hebrew University-Hadassah Medical School, Jerusalem 9222507, Israel; elliotb@ekmd.huji.ac.il

**Keywords:** sustainable food systems, Mediterranean Diet, Global Nutrition Index

## Abstract

During the past decade, the concept of sustainability has been added to the factors involved in food security. This has led to a more comprehensive and holistic approach to sustainable food systems which considers drivers—environment, geopolitics, demographics, policy regulations, socio-cultural-economic factors, science and technology and infrastructure. The outcomes, similarly, involve many dimensions—environment, food security and nutrition, health and socio-cultural-economic aspects. This article discusses the Mediterranean diet in the context of sustainable food systems and shows (as in all parts of the world) that there is food insecurity in every country as monitored by the Global Nutrition Index. Three recent, major reports published in 2019 suggest what measures need to be taken to improve sustainable food systems. All environmental analyses agree on the need to promote more plant-based diets—achieved practically by using “more forks than knives”. The Mediterranean Diet pattern is a case study for a sustainable diet. It has the best scientific evidence for being healthy, together with economic and socio-cultural benefits. A major challenge is that it is not consumed by the majority of the population in the Mediterranean region, and any solution must involve equity—the socially just allocation of resources. The task now is implementation with multi-stakeholder involvement, in the knowledge that “a well fed nation is a healthy nation is a sustainable and productive nation”.

## 1. Introduction

The nine major areas of nutrition action selected by the FAO/WHO following the international conference on nutrition, 1992, “Nutrition and development: a global challenge” [1] were 1. Improving household food security. 2. Preventing micronutrient deficiencies. 3. Preventing and managing infectious diseases. 4. Promoting healthy diets and lifestyles. 5. Enhancing the capacity for care. 6. Improving food quality and safety. 7. Assessing, analyzing and monitoring nutrition situations. 8. Incorporating nutrition objectives into development policies and programs and 9. Promoting breast-feeding.

We may ask what has changed over the past twenty-five years to these objectives. First there was the addition of Stability as a fourth dimension to food security in the wake of the world financial and food crisis of 2008. Secondly, has been the promotion of <sustainability> as in the concepts of <sustainable diets> and <sustainable food systems> [2,3]. In some aspects, sustainability may be considered as the fifth long-term time dimension to food security incorporating social-cultural, environmental and economic welfare for future generations [4]. Finally, there is the recognition that practical attainment of such essential goals requires multi-stakeholder involvement and expansion of food systems to deal with geo-political challenges posed by the progressive urbanization of the world population and man-made and natural disasters. This means considering representatives from agriculture (farmers and global business), nutrition, health, emergency preparedness, economics, education, academia, food industry, retailers, restaurant chains, street vendors, non-governmental organizations (NGOs) and civil society (consumers), law, policy makers (government, local authorities) and more.

The resulting synthesis has been discussed recently in three summary publications [5,6,7]—the Lancet EAT commission on healthy diets from sustainable food systems; the international expert report on the policy reform and realignment that is required to build sustainable food systems in Europe; and the FAO report on biodiversity for food and agriculture. Of interest, the last two reports make no mention of the Mediterranean diet even though it has been shown to exhibit major sustainability benefits [8].

## 2. Sustainable Food Systems

A Sustainable Food System “is a food system that ensures Food Security and Nutrition (FSN) for all in such a way that the economic, social and environmental bases to generate FSN of future generations are not compromised” [9].

According to the Global and Environmental Change and Food Systems project [10], Food Systems encompass: (1) activities related to the production, processing, distribution, preparation and consumption of food and (2) The outcomes of these activities contribution to food security: *Food availability*, with elements related to production, distribution and exchange; and *Food access*—affordability, allocation and preference and *Food use*—nutritional value, social value and food safety. These outcomes also contribute to environmental and other securities (e.g., income, health). Interactions between and within bio-geophysical and human environments influence both the activities and drivers and the outcomes [11] (Table 1 and Table 2).

The drivers of food systems are shown in Table 1.

The outcomes of food systems relate to four principal areas: human health and wellbeing; ecological health and wellbeing; economic prosperity and social equity [12].

## 3. Equity

Whereas equality denotes the highest average level of a <good>, equity refers to the “socially just” allocation of that <good>. In practical terms, this means dealing with a number of areas: 1. Providing fair access to universal public services: Maternal and infant health, immunization and sanitation. 2. Action for disadvantaged food insecure groups: Socio-cultural: (ethnicity, gender), immigrants, homeless, institutionalized people, pregnant women, children and the elderly. 3. Social protection: nutritional safety nets—cash, stamps, food. This may be conditional on compliance with public health and societal goals such as immunizing children and making them attend schools. 4. Redistribution of goods and services: Land reform women’s empowerment, sovereignty issues, fiscal trade policies for affordable nutritious foods, taxes from “unhealthy” foods re-invested in food system. 5. Challenging imbalances of power through civil society and independent media: This covers conflicts of interests between multinational agro-business and. small farmers, interests of food producers versus those of consumers, accountability and the demands of civil society [13].

**Figure 1:**
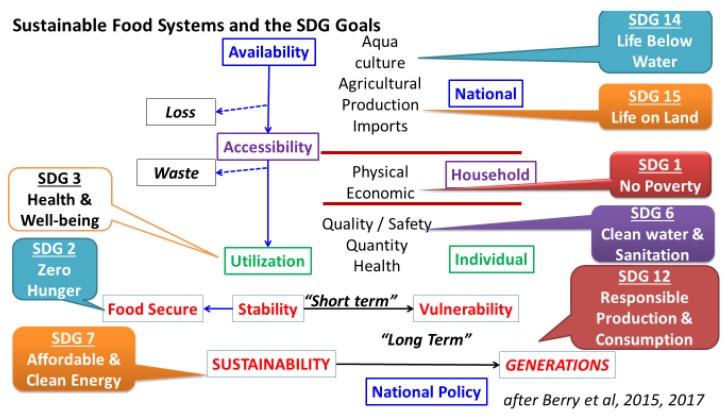
The relationships between the food security pathway and many of the sustainable development goals.

The items listed as drivers and outcomes of food systems activities are not exhaustive but serve to highlight the multidisciplinary factors that are involved and why multi-stakeholder involvement is so important to ensure efficient and successful systems. It is therefore not surprising that many of the Sustainable Development Goals (SDG) relate to the food security pathway—availability, accessibility, utilization and stability—and sustainable food systems. Figure 1 shows the relationships between the food security pathway and eight of the SDGs. As an exercise, it is also possible to position *all* the 17 SDGs to food security and nutrition within the framework of sustainable food systems [14].

## 4. Malnutrition in Mediterranean Countries

The Mediterranean Diet has been championed as a case study for sustainable diets as it embodies the four above outcomes [8,15]. However, this is does not mean that the Mediterranean area does not have nutritional problems similar to other developed countries, although there is a marked north-south divide. We have developed a global nutritional index (GNI) to capture the triple burden of malnutrition—hidden hunger (micronutrient deficiency), protein-energy malnutrition and obesity. The higher the GNI the better the nutrition status and the less malnutrition [16].

**Figure 2:**
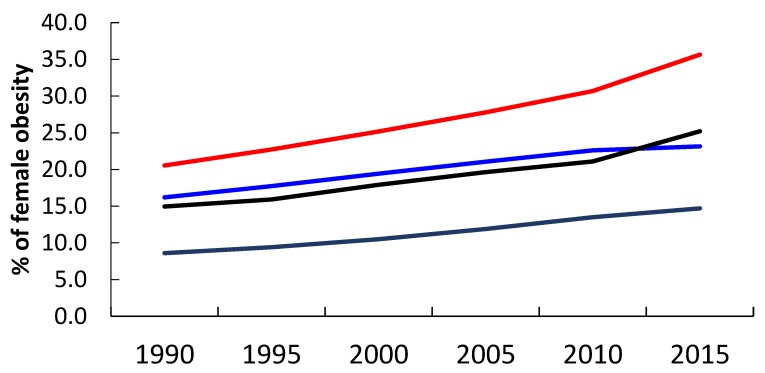
Percent of female obesity (BMI >30 kg/m^2^) in Mediterranean country groups, 1990–2015, compared to world trends. Key (from top to bottom): Red line North African and Eastern Mediterranean countries; Blue line High Income countries; Black line Eastern Europe LMIC; Grey line World. (Source reference 16 from WHO data).

According to the World Bank and WHO, the twenty countries bordering the Mediterranean may be classified into three groups: North African and Eastern Mediterranean Low-Middle Income Countries LMIC (7) Algeria, Egypt, Lebanon, Libya, Morocco, Syria, Tunisia; European LMIC (4) Albania, Bosnia and Herzegovina, Montenegro, Turkey, and the rest High Income Countries (9) Croatia, Cyprus, France, Greece, Israel, Italy, Malta, Slovenia, Spain. Time trends from 1990 to 2015 show that obesity in all the regions has increased steadily (Figure 2).

The ranges for GNI in 2015 according to the county groups were as follows: high-income countries from 0.724 (Italy) to 0.576 (Croatia); for the European LMIC, from 0.613 (Bosnia and Herzegovina) to (Turkey) (0.466). For North Africa and East Mediterranean LMIC, the indices ranged from 0.531 (Tunisia) to 0.377 (Egypt). The purpose of GNI is not to “name and shame”, but, rather, to learn from other similar countries how to improve the situation. In summary, since 1995 the triple burden of malnutrition has shown a shrinking hungry, and expanding fat world. In the Mediterranean Countries, the GNI has in fact decreased, except for European LMIC. The plea is to return to traditional Mediterranean lifestyle choices of frugality and moderation at meal times.

## 5. Environmental Impact of the Mediterranean Diet

Global environmental changes include transformations in the physical and bio-geochemical environment, either caused naturally or by human activities such as deforestation, fossil fuel consumption, urbanization, land reclamation, agricultural intensification, freshwater extraction, fisheries over-exploitation and waste production. These have effects on global warming and atmospheric composition, land cover and soils, climate variability, water availability and quality, nitrogen availability and cycling, sea levels, currents and salinity and biodiversity on land and in the sea. Biodiversity is reflected at three levels: the ecosystem or agro-ecological zone, the species contained in the ecosystem and the genetic diversity within the species [17].

Data from the Global Footprint network [18] show that the ecological footprint of Mediterranean countries is higher in HIC countries. The principal drivers are Food (35%), Transportation (28%) and Housing (9%). The share of the food footprint ranges from 20% (Slovenia) to 70% (Morocco). Egypt and Slovenia have a high calorie and low footprint due to a low protein diet and high crop productivity, with a low dependence on imported food. Conversely, Portugal and Malta have a high footprint due to a protein-intensive diet rich in fish (44%) and meat products (16%).

Since food is one of the key human requirements, resource needs can only be altered to a small extent. Population growth, urbanization and a shift towards protein-based, energy rich diets will increase globally adding pressure on ecosystem services. Thus, future food security depends on efficiency improvements, reduction of food waste and promotion of healthier and less resource-intensive diets. The added value of the Mediterranean diet is that it has many typical *local* products (e.g., oil, vegetables) produced by small and medium enterprises. There is a balanced food supply, less carbon-intensive mechanized agriculture and good health quality of food. Valorizing the Mediterranean diet depends on food quality control, traceability (reduced producer–consumer distance), and increased market opportunities for small local, traditional producers.

Calculations show that changing to a Mediterranean dietary pattern in Spain would reduce the environmental impact (−72%), land use (−58%), energy (−52%) and water (−33%) consumption [19]. However, similar changes in eating patterns may have variable environmental influences. Consider two scenarios that both lead to a nutritious diet and healthy caloric intake. One: Moderate meat, high in dairy, high in fruit and vegetables from green houses or airfreighted, high in fruit and vegetables grown in water stressed regions. Two: Low meat, moderate dairy, high in legumes and pulses, high in seasonal fruit and vegetables. Scenario “One” increases the environmental impact, whereas scenario “Two” will decrease it. Attempts to follow Mediterranean dietary patterns and balance financial costing can be complicated as in the case of oranges consumed in the UK. Most of these surprisingly come from Brazil, rather than from the Mediterranean, and are shipped across the Atlantic, but still have low carbon footprints. This is because 60 per cent of an orange’s life-cycle footprint has been embedded in their flesh before it has left the farm gate [20]. Most of this comes from fertilizers, pesticides and the fuel used by machinery during harvesting. The oranges are then processed and sorted. If the orange is used to make juice, 22 per cent of the total footprint is down to transport and distribution.

According to UK government data, buying a tomato grown locally has three times the footprint of a tomato grown in Spain. Similarly, lettuces grown during winter are cultivated in poly tunnels, which require much energy to keep them warm. In terms of carbon emissions, it is more environmentally friendly to buy them from Spain during the winter and locally during the summer.

However, food miles do matter if the product has been transported by air. For this reason, consumers should avoid eating out-of-season soft fruit such as raspberries and blueberries. A 100 g box of blueberries grown locally or imported via ship will produce around 100 g of carbon dioxide. If the produce is flown in, that increases by ten times, pushing its carbon footprint up to more like 1 kg. However, such calculations are more complex, especially if one looks at only carbon emissions. In contrast, ships use dirty fuels that emit nitrogen and sulphur as well as carbon dioxide, which has a significantly larger greenhouse gas impact than carbon dioxide alone. Food systems contribute to between 20 and 30 per cent of emissions globally. Eating vegetarian one day a week could reduce a person’s overall impact by around 4 per cent, especially if the produce is in season.

The EAT-Lancet commission report recommends a much more comprehensive classification of the environmental effects of foods by listing five measurements per serving. Greenhouse gases (g CO_2_ equivalent); Land use (m^2^); Energy use (Kj); Acidification potential (g Sulphur dioxide-equivalent) and Eutrophication (g Phosphate-equivalent) [5]. Eutrophication is excessive richness of nutrients in a lake or other body of water, frequently due to runoff from the land, which causes a dense growth of plant life, particularly algae, and death of animal life from lack of oxygen. Two additional measures that should be factored into calculations of environmental effects are nitrogen application and water use. Each country must also adopt an integrated water management and conservation policy. This should include desalination by reverse osmosis; drip irrigation and reusing treated sewage for farming; engineering crops to thrive in onerous conditions; discouraging large domestic gardens; finding and fixing leaks early; and making efficient toilets mandatory with double flushing systems.

Animal source foods account for some 75% of climate change effects, whereas staple crops such as wheat, rice and other cereals are responsible for 30%–50% of pressures on other environmental domains [5]. From all these indicators, the consensus is that plant-based diets have the least environmental impact and could release up to 76% of the land currently used for farming (See also [21]). This land could then be used for the mass restoration of ecosystems and wildlife [22]. These data show that dietary change can deliver environmental benefits on a scale not achievable by producers. Moving from current diets to a diet that excludes animal products has transformative potential, reducing food’s land use by 3.1 (2.8–3.3) billion ha (a 76% reduction), including a 19% reduction in arable land; food’s GHG emissions by 6.6% (5.5–7.4) billionmetric tons of CO_2_ eq (a 49% reduction); acidification by 50% (45%–54%); eutrophication by 49% (37%–56%); and scarcity-weighted freshwater withdrawals by 19% (−5%–32%) for reference year 2010 [22]. However, these calculations are rather utopian, since animal-based foods cannot be eliminated worldwide. In addition, they undervalue the nutritional advantages of eggs (for example) as a biologically rich and cheap source of first class protein.

## 6. Practical Actions towards Sustainable Food Systems

What are countries doing? A recent study of guidelines for sustainability from eleven countries —not from the Mediterranean region (but applicable to it also)—listed some thirteen points. The three most recommended were (1) more plant foods (9 countries), (2) reduce food waste (7 countries) and (3) eat less meat (5 countries) [23]. These may be summarized in advice to “use more forks than knives”.

An original attempt to reduce the use of pesticides is biological pest control as practiced in the Middle East among Israeli, Jordanian and Palestinian farmers. “Nature to fight nature” with barn owls and kestrels to control the rodent populations [24].

Future trends will involve a moderate reduction in meat and dairy consumption, and developing alternative sources of protein such as from insects (where culturally acceptable) or from laboratory-grown meat.

The case of fish is of particular interest. Environmental effects can differ greatly between captured and farmed fish. Nutrient content will depend on what the fish eat or are fed. Finally, there is problem of insufficient supply versus the demand to follow nutritional and health recommendations to eat two fish servings per week [25]. Therefore, alternative sources for dietary omega-3 fatty acids are required.

## 7. National Recommendations for Sustainable Food Systems

The following are a list of core recommendations made at a conference on Sustainable food systems, held in Tel Aviv in 2016 [26].

1. Make Food System Sustainable along the entire food chain—from Production to Consumption; reduce food losses and waste. Involve multi-stakeholder partners.
2. Strengthen Agriculture towards the best Sustainable Ecosystem practices.
3. Protect the Right of All to healthful, nutritious, adequate and affordable food.
4. Monitor the safety of the food supply to be environmentally friendly and free of pathogens.
5. Legislate (and incentivize) the Food Industry to produce healthy, (minimally processed foods), with less added sugars, salt and additives. Informative Labelling with consumer messages as in “traffic light”. Production and Marketing must be honest and transparent. No junk food adverts to children.
6. Promote School and Community Education on healthy life styles, nutrition, cooking (Mediterranean Diet Pattern) and exercise. Lifestyle friendly environments with shade, water stations, bicycle lanes. Encourage community gardens and farmer’s markets.
7. Promote access of healthy foods in restaurants, public places, hospitals, universities and in office canteens.

In connection with the last point, we have recently completed a study showing that it is possible to change successfully the eating habits of a kibbutz community by an intervention in the main dining kitchen towards the Mediterranean dietary pattern [27,28].

## 8. Regional Policy

With regard to the recent report for the EU [7], five objectives of a common food policy are recommended to provide a new governance architecture for sustainable food systems: 1) ensuring access to land, water and healthy soils; 2) rebuilding climate-resilient, healthy agro-ecosystems; 3) promoting sufficient, healthy and sustainable diets for all; 4) building fairer, shorter, and cleaner supply chains and 5) putting trade in the service of sustainable development. The documentation includes some 477 references and one impossible figure, number 11, which is so complicated that it requires an aspirin and wet towel to study. However, there are some keen insights such as: “Building healthy food environments requires a fundamental rethink of urban development, transport, and mobility systems; and it requires social policies that unite citizens across economic divides and make a healthy and meaningful life possible for all. Ultimately, the prevailing incentives to over-produce, to over-consume, and to externalize costs onto taxpayers and future generations must be replaced by a new green taxation paradigm, and by a macro-economic paradigm no longer focused on GDP growth as an end in of itself. These changes are civilizational in nature. They must be underpinned by a new contract between citizens, businesses, and policymakers. The governance for transition described in this report therefore does not only apply to food systems. The sustainability challenge is cross-cutting, and the solutions must be too”.

## 9. Conclusions

Not all food-secure diets are sustainable, but all sustainable diets should be food-secure. Sustainable Food Systems are beneficial for every citizen, for every country and thus good for the planet—but everyone must play their part. This will require adopting a healthy diet, making determined efforts to reduce food losses and waste and enhancing food production by both agriculture and industry. Shifting food systems towards more sustainability requires joint action from consumers, academia, stakeholders and policy makers. There are still many research gaps regarding sustainable diets especially concerning implementation see [29] and articles therein.

While the food industry may be part of the problem, it must also be part of the solution. Potentially, the best evidence-based, healthy, sustainable diet is the Mediterranean Dietary Pattern [15]. The challenge is how to make this the dominant dietary lifestyle for the majority of the people living in the Mediterranean region. Another question is whether this pattern is applicable to other regions of the world. While the composition of the Mediterranean diet and lifestyle is relatively standard—based on olive oil, grains, pulses fruits and vegetables, and little processed foods, there are necessarily *country-specific variations* in sustainable eating patterns incorporating these ingredients, according to local cultures and traditions [8]. These, of course, should be preserved and promoted, together with the conviviality aspects of meal times. It is salutary to remember that a healthy diet is not just a list of “do’s” and “don’ts”, but rather should be a pleasurable, social and tasty experience. Finally, as for most lifestyle behaviors, the earlier they are taught and practiced, the more likely they are to take root and persist [30]—as has been said “Train up a child in the way he should go: and when he is old, he will not depart from it”. (Proverbs 22:6)

## Figures and Tables

**Table 1:** The various drivers of Food System Activities.

Food System Drivers	Examples
**Environment**	Natural resources, Biodiversity, Ecosystem services, Climate change
**Geopolitics**	Political stability, Leadership, Globalization, International trade, Conflicts and humanitarian crises Food policies, Food prices and volatility
**Demographics**	Population growth, Changing age distribution Urbanization, Migration and forced displacement
**Policy Regulations**	Taxes and subsidies, Land rights/tenure
**Socio-Cultural, Economic**	Education, Gender inequalities, Women’s empowerment Health, Market opportunities, Income distribution Societal values, Traditional knowledge, Religion and Rituals
**Science Technology**	Research and Development, Innovation, High-Tec, Information
**Infrastructures Institutions**	Roads, Ports, Transport, Energy grids, Government, Food industry, Companies, NGOs, Civil society

**Table 2:** Four Food System outcomes.

Food System Outcomes	Examples
**Environment**	Resource efficiency, Conservation and sustainable biodiversity Ecosystem services, Climate change mitigation
**Food Security and Nutrition**	Availability → Accessibility → Utilization → Stability → Sustainability, Minimizing Food loss and waste
**Health**	Human capital, Employment Productivity
**Social-Cultural-Economic**	Poverty alleviation, Livelihoods living wages, Social justice, Equity, Resilience, Advocacy, Trust
